# SodiuM glucose cotrANsporter-2 (SGLT-2) inhibitors in the treatment of type II DiAbetes in Tuscany: utiLizatiOn patteRns, and relatEd clinical use evaluation; the MANDALORE study protocol

**DOI:** 10.3389/fendo.2026.1741084

**Published:** 2026-03-23

**Authors:** Irma Convertino, Ersilia Lucenteforte, Janette Monzillo, Silvia Velo, Emiliano Cappello, Graziano Di Cianni, Giuseppe Penno, Matteo Fornai

**Affiliations:** 1 Unit of Pharmacology and Pharmacovigilance, Department of Clinical and Experimental Medicine, University of Pisa, Pisa, Italy; 2 Tuscan Regional Center of Pharmacovigilance, Florence, Italy; 3 Department of Statistics, Computer Science, Applications “G. Parenti”, University of Florence, Florence, Italy; 4 Diabetology and Metabolic Diseases Unit, Azienda USL Toscana Nord-Ovest, Livorno Hospital, Livorno, Italy; 5 Metabolic diseases and Diabetology/Endocrinology Unit, University Hospital of Pisa, Pisa, Italy

**Keywords:** Type 2 diabetes mellitus, SGLT2 inhibitors, gliflozins, drug utilization, pharmacovigilance, diabetic ketoacidosis, adverse drug reactions, real-world data

## Abstract

**Background:**

Diabetes mellitus is a globally prevalent disease, with an incidence of 5–7 new cases per 1000 person-years and no significant gender disparity. Sodium-glucose co-transporter 2 (SGLT-2) inhibitors are recommended as second-line therapy after metformin in uncomplicated type 2 diabetes, whereas they are recommended as first-line treatment in patients with heart failure or other cardiorenal comorbidities. Given the recent introduction of SGLT-2 inhibitors, their increasing utilization, and the heterogeneity of existing literature, real-world evaluations of these agents are warranted.

**Objectives:**

The MANDALORE study, a regional active pharmacovigilance project approved by the Italian Medicines Agency (AIFA), aims primarily to assess the drug utilization of gliflozins in clinical practice. The secondary objective is to analyze safety outcomes and investigate the determinants of treatment switching events.

**Methods:**

This retrospective cohort study will analyze medical records of patients who underwent at least one diabetes visit at the Metabolic Diseases and Diabetology/Endocrinology Unit of Pisa University Hospital and the Diabetology and Metabolic Diseases Unit of Livorno Hospital. Inclusion criteria comprise that Tuscan patients receiving their first prescription for a gliflozin (index drug) between 2012 and 2021 (index date). Outcomes to be assessed include treatment switching, time to switching, line of therapy, dose adjustments, and time to dose change. Gliflozin utilization will be investigated, particularly in relation to concomitant interacting or contraindicated medications, and in patients requiring caution as specified by AIFA labeling and relevant tools. Incidence rates will be calculated for secondary safety outcomes, including adverse drug events (ADEs) leading to emergency room visits or hospitalization, non-serious ADEs, and specific ADEs as diabetic ketoacidosis, urinary and genital infections, gangrene, lower limb amputations, and neoplastic events. ADEs and therapeutic effectiveness observed in proximity to switching events will be investigated.

**Results:**

The findings from the MANDALORE study are expected to elucidate critical aspects of gliflozin use in routine clinical practice, particularly within the Tuscan regional healthcare context, thereby providing valuable insights from the real-world for clinicians and healthcare systems.

## 1 Introduction

Diabetes mellitus is a major global health condition, affecting an estimated 537 million adults worldwide in 2021, with projections reaching 643 million by 2030 and 783 million by 2045 (1). In Italy, in 1985, there were approximately 1.5 million known cases of diabetes and, in 2015, cases reached 4 million, with 250,000 new diagnoses of diabetes per year (2). According to the Italian National Institute of Statistics (ISTAT), the prevalence of diagnosed diabetes in Italy was estimated at 6.2% in 2023 (6.9% in men and 5.7% in women, crude rate), corresponding to nearly 4 million individuals. Prevalence increases markedly with age, reaching approximately 20% among individuals aged 75 years or older. Regional differences are observed, with lower prevalence in Northern regions and higher rates in Southern Italy and the Islands. Over the past decade, a gradual upward trend has been reported, with a slight decrease in 2023. Type 2 diabetes accounts for approximately 90% of all diabetes cases and is strongly associated with overweight/obesity, unhealthy diet and sedentary lifestyle. The increase in diabetes prevalence has been mainly attributed to the growing diffusion of these risk conditions in the population (3).

The treatment of type 2 diabetes mellitus includes metformin as first-line therapy. In cases of marked hyperglycaemia or the presence of classic symptoms of diabetes, initial combination therapy with metformin and another agent may be considered, even in drug-naïve patients. If metformin monotherapy is insufficient to achieve adequate glycaemic control, a second agent should be added. If dual therapy remains inadequate, triple therapy is recommended. When evaluating efficacy, safety, and tolerability, drugs that may be combined with metformin include pioglitazone, dipeptidyl peptidase-4 (DPP-4) inhibitors, glucagon-like peptide-1 (GLP-1) receptor agonists, and sodium–glucose cotransporter-2 (SGLT2) inhibitors. The choice of the second agent should be tailored to individual patient characteristics, taking into account comorbidities as well as the risks and benefits of each drug. In obese patients, agents that do not promote weight gain, such as metformin, DPP-4 inhibitors, GLP-1 receptor agonists, and SGLT2 inhibitors, should be preferred. Glibenclamide is associated with a higher risk of hypoglycaemia compared with other sulfonylureas. When sulfonylurea therapy cannot be avoided, gliclazide is preferable because of its more favorable safety profile, particularly regarding hypoglycaemia and cardiovascular risk. In patients with a history of major cardiovascular events, the preferred first-line add-on agents are long-acting GLP-1 receptor agonists, SGLT-2 inhibitors, and pioglitazone, unless contraindicated. Insulin therapy should be initiated when glycaemic control with non-insulin agents, even in combination therapy, remains unsatisfactory. Insulin treatment, even if temporary and with or without metformin, may be considered at any time during the course of the disease in the presence of marked metabolic decompensation or severe symptoms of hyperglycaemia. Long-acting analogues are preferable to human insulin, and rapid-acting analogues are preferable to regular human insulin. When not contraindicated, metformin should be continued after insulin initiation. Insulin doses should be titrated according to blood glucose levels until target values are achieved. Adding SGLT-2 inhibitors, GLP-1 receptor agonists, or DPP-4 inhibitors to insulin therapy, with or without metformin, may help limit daily insulin requirements and reduce weight gain (4).

In Italy, the following agents have been approved: canagliflozin (2013), dapagliflozin (2012), empagliflozin (2014), and ertugliflozin (2018). Between 2012 and 2021, the positioning of SGLT2 inhibitors in the treatment of type 2 diabetes mellitus was progressively redefined. In the Italian Standards of Care for Diabetes (2014 and 2016 editions), SGLT2 inhibitors were recommended as second- or third-line options after metformin, primarily for glycaemic control, alongside other agents such as DPP-4 inhibitors, pioglitazone, sulfonylureas, or insulin (5, 6). From 2015 onwards, major cardiovascular and renal outcome trials demonstrated that, beyond HbA1c reduction, SGLT2 inhibitors reduced major cardiovascular events, hospitalizations for heart failure, and progression of diabetic kidney disease (7–9). These findings led to a revision of their therapeutic positioning, with preferential use in patients with established cardiovascular disease, heart failure, or chronic kidney disease, including at earlier stages of the treatment algorithm, either in combination with or as an alternative to metformin (10). As reported in the OSMED 2019 report published by the Italian Medicines Agency (AIFA), the use of gliflozins has increased over time (11).

Evaluating the utilization of SGLT-2 inhibitors would allow a better understanding of their use in clinical practice and provide insight into adherence patterns. As shown in previous Tuscan real-world analyses of chronic therapies, persistence and discontinuation analyses can help characterize treatment dynamics in routine care (12, 13).

In 2011, the Food and Drug Administration (FDA) issued an increased risk of cancer bladder and breast linked to the use of dapagliflozin (14). Furthermore, in the literature the risk of bladder cancer has also been associated with patients treated with empagliflozin (7). A risk of diabetic ketoacidosis, a state of metabolic acidosis with increased anion gap and ketonemia/ketonuria, which, unlike classic ketoacidosis, is not characterized by a state of hyperglycaemia but of euglycemia, was observed by the use of gliflozins (15). A study using the adverse event reporting system of the FDA highlighted 680 cases of ketoacidosis among 5,694 patients treated with dapagliflozin (11.9%), 1,362 cases of ketoacidosis among 14,117 patients treated with canagliflozin (9.6%) and 355 cases among 2,719 patients treated with empagliflozin (13.1%), while only 1.34% of patients treated with other oral antidiabetic drugs developed ketoacidosis (16). In patients with type 2 diabetes mellitus, who already have an increased risk of genitourinary infections, the use of SGLT2 inhibitors further increases this risk (17–19). In the CANVAS study infections at the genital level were highlighted in both men and women (in this latter fungal infection), treated with canagliflozin, while no significant differences were observed in case of urinary tract infections between treated and placebo (8). In literature, only dapagliflozin is associated with an increased risk of genitourinary infections (20). The CANVAS study showed an increased risk of amputation of toes, feet and legs in subjects treated with canagliflozin (8). This risk, however, was not observed with empagliflozin in the EMPA-REG OUTCOME study (7).

In conclusion, the increasing use of SGLT2 inhibitors, together with the heterogeneity of evidence reported in the literature, highlights the need for real-world studies to better characterize their role in routine clinical practice. This project is aimed at identifying the usage profile and evaluating the concomitant interacting drugs and the use in patients requiring caution. Adverse events (AEs) of interest, including genitourinary infections, diabetic ketoacidosis, acute kidney injury, and other clinically relevant outcomes, will be analyzed.

## 2 Materials and methods

This study protocol was written according to the ICH M14 Guideline *“General Principles on Planning, Designing, Analyzing, and Reporting of Non-interventional Studies That Utilize Real-World Data for Safety Assessment of Medicines” (*21).

### 2.1 Study design and setting

This study will be a population-based retrospective observational study with a case monitoring cohort design. The MANDALORE study will be conducted on data from the Metabolic Diseases and Diabetology/Endocrinology Unit of the University Hospital of Pisa and the Diabetology and Metabolic Diseases Unit of the Livorno Hospital. The Unit of Pharmacology and Pharmacovigilance of the Department of Clinical and Experimental Medicine of the University of Pisa affiliated with the Departmental Section of Adverse Drug Reactions Monitoring of the University Hospital of Pisa will coordinate the study. The study will be carried out from September 2023 to March 2027, and it will use data collected from the earliest available data to December 31, 2025 (study period). The study will have a total duration of 42 months. Data extraction and analysis processes will have a total duration of 15 months (October 2025-December 2026). A final period of three months will be dedicated to the finalization of the study (final report and dissemination of results).

### 2.2 Healthcare setting and regulatory framework

During the inclusion period (1 January 2012–31 December 2021), SGLT2 inhibitors were initiated by authorized specialists within the Italian National Health Service (Servizio Sanitario Nazionale, SSN). Prescriptions were issued using standard SSN prescriptions. After specialist initiation, therapy could be continued through SSN prescriptions also by general practitioners, within the regulatory framework in force at the time. Drug dispensing occurred either through hospital-based direct distribution or through community pharmacies under regional distribution agreements. Follow-up was conducted in outpatient specialist care as part of routine diabetes management.

Regulatory conditions were subsequently modified after the inclusion period, further expanding prescribing autonomy within the SSN; however, these changes fall outside the timeframe considered in the present study.

### 2.3 Data sources

We will use data included in the patient medical records of these wards. Information regarding demographic data (age, gender), medication prescriptions (drugs and dosages), adverse events recorded, specialist visits, and laboratory parameters documented in the patient record (HbA1c, eGFR) will be extracted. These records reflect information routinely documented within the participating Diabetology Units as part of standard clinical care. While they provide detailed data on diabetes management within these centers, access to general practitioner records or to medical records from other hospital departments or external healthcare facilities is not systematically available, unless such information is reported in the specialist medical record. Consequently, clinical events or comorbidities managed outside the participating centers may not be fully captured. Detailed description of the variables is available in the **Supplementary Material**. The evaluation will include both inpatient and periodic outpatient specialist visits. A preliminary assessment of accesses to these wards showed approximately 10,000 patients. Patients who will accept to participate in the study will sign the informed consent.

### 2.4 Study population

#### 2.4.1 Inclusion and exclusion criteria

Residents in Tuscany with the first supply of canagliflozin, dapagliflozin, empagliflozin, ertugliflozin (SGLT-2 inhibitors) (index drugs) between 2012 – 2021 (inclusion period) and at least one diagnosis of diabetes will be included and the date of first prescription of these drugs will represent the index date. The drugs will be identified by the Anatomical Therapeutic Chemical (ATC) code or description, diagnoses by the International Classification of Diseases, 9th revision (ICD-9) codes or description. Patients will be followed from the index date until the event of interest, death, loss to follow-up, or end of study.

Patients were excluded if they were not residents in Tuscany at the index date, had no documented diagnosis of diabetes, had received an SGLT2 inhibitor in the 12 months prior to the index date (i.e., not new users), had an index date outside the inclusion period, or had insufficient data to reliably define exposure or assess study endpoints.

#### 2.4.2 Endpoints and exposure

In the primary endpoints, the exposure will be defined in a time-dependent manner. Patients will be considered exposed to the index drug until documented discontinuation or therapeutic switch (i.e., replacement of the index drug). The introduction of an additional antidiabetic agent without discontinuation of the index drug will be classified as add-on therapy, and person-time will continue to be attributed to the index drug. A gap of more than 6 months without prescription renewal will be considered treatment discontinuation in the primary analysis. From the date of interruption, person-time will no longer be attributed to the index drug. If treatment is re-initiated after such a gap, a new exposure episode will be defined. A sensitivity analysis will be performed considering a discontinuation gap of more than 12 months.

In this way, each patient will be able to contribute person-time to different exposure groups.

Primary endpoints:

-Description of patients with switching within the entire gliflozin class and by individual drug (alone or in combination with metformin and/or gliptins)
-Description of patients by treatment regimen within the entire gliflozin class and by individual drug (alone or in combination with metformin and/or gliptins)
-Description of patients by dose change within the entire gliflozin class and by individual drug (alone or in combination with metformin and/or gliptins)
-Time to introduction of a new antidiabetic agent (treatment intensification/add-on or switching)
-Time to the first dose change
-Identification and description of patients using gliflozin and concomitant interacting and/or contraindicated medications
-Identification and description of patients who will be classified as users in whom gliflozin use should be approached with caution

Secondary Endpoints:

The incident rate for the following adverse events will be assessed:

-Events that resulted in emergency room visits and all-cause hospitalizations.
-Events that did not result in emergency room visits and all-cause hospitalizations.

Acute specific events:

-Diabetic ketoacidosis
-Urinary and genital infections
-Gangrene
-Lower limb amputation

Long-term specific events:

-Tumors

For the tumor endpoint, a 2-year lag period will be applied (the patient will be considered exposed only 2 years after the first administration of the index drug). A sensitivity analysis will also be performed, reducing the lag period to 1 year. The most common types of tumors will be described. In the event of a therapeutic switching from a first to a second study drug, the patient will contribute person-time attributed to the first drug for the entire duration of the lag period, and all events occurring during the lag period will be attributed to the first drug. Patients with a previous diagnosis of cancer in the 5 years before the index date will be excluded or stratified in subgroups (yes previous cancer diagnosis vs no previous cancer diagnosis) based on the numerosity retrieved.

Time free from the above-mentioned adverse events will also be assessed.

The following events will be assessed in patients switching:

-Adverse events
-Effectiveness (HbA1c, eGFR)

In patients experiencing adverse events, therapeutic management will be described, including treatment discontinuation, dose reduction, continuation without modification, and possible re-initiation of the index drug, when such information is available in the clinical documentation. Specifically, these events will be considered potentially related when recorded in the switching time frame (time related).

The analysis will also be stratified by drug to highlight differences between users of individual formulations.

### 2.5 Data analysis

The baseline characteristics of the included cohort will be described using descriptive statistical tests in terms of age, sex, previous history of diabetic disease, number of concomitant medications (defined as all pharmacological therapies documented in the electronic medical records, irrespective of prescription status), other antidiabetic medications, concomitant pathologies, and year of cohort entry.

#### 2.5.1 Primary endpoints

Treatment regimens, switching, and dose changes in gliflozin treatments (by class and by formulation) will be analyzed for each patient with at least two prescriptions. The time to introduction of a new antidiabetic drug will be analyzed using the Kaplan-Meier method by calculating survival curves from the index date to the date of introduction of the new antidiabetic drug (switching) and the time to the first dose change.

Patients will also be classified based on the use of concomitant medications classified as interacting and/or contraindicated and/or to be used with caution. These classifications will be performed based on gliflozin prescriptions that meet the requirements of the AIFA technical data sheet (22) and specific tools. Baseline characteristics will be described, and differences will be tested.

#### 2.5.2 Secondary endpoints

The incident rate for all safety events will be reported.

The time free from the safety events will be analyzed using the Kaplan-Meier method by calculating survival curves from the index date to the date of the event of interest.

Safety and effectiveness events plausibly related to the switching events will be reported.

If numerosity will allow, descriptive analyses will be conducted for subgroups of patients exposed to gliflozins based on their formulation (i.e., single or in combination), and tested for differences.

Only for the long-term cancer safety events’ assessment we will stratify or exclude (according numerosity) the patients with a previous diagnosis of cancer in the 5 years before the index date.

These data will be elaborated by using Stata Statistical Software v14 (Stata Corporation, College Station, TX). In **Table 1** is displayed a brief overview of the data analyses.

**Table 1:** Summary of analyses.

Objectives	Measurements	Analyses
Drug-utilization (primary endpoints)	Patient description by treatment line, switching and dose change	Descriptive analysis
	Time to introduction of a new antidiabetic (switching), and to change dosage	Kaplan Meier
	Identification and description of patients with concomitant medication use classified as interacting, contraindicated, and to be used with caution	Descriptive analysis
	Identification and description of patients classifiable as gliflozin users with caution	Descriptive analysis
Safety (secondary endpoints)	Adverse events that resulted in hospitalizations and emergency room visits for all causes and events that did not result in hospitalizations and emergency room visits for all causes	Incident rate and descriptive analysis
	Time free from the first event that led to access to the emergency room and/or hospitalization for all causes and from the first event that did not lead to hospitalization and access to the emergency room for all causes	Kaplan Meier
	Adverse events specific to ketoacidosis, urinary and genital infections, gangrene, lower limb amputation and tumours	Incident rate and descriptive analysis
	Time free from the first event of ketoacidosis, urinary and genital infections, gangrene, lower limb amputation and tumours	Kaplan Meier
	Effectiveness and safety events detected in proximity to switching events	Incident rate and descriptive analysis
	All indicators will also be evaluated for each individual drug (where numerosity will allow it)	Descriptive analysis/incident rate/Kaplan Meier

## 3 Data management

The data extracted from the patients’ medical records of the Metabolic Diseases and Diabetology/Endocrinology Unit of the University Hospital of Pisa and the Diabetology and Metabolic Diseases Unit of the Livorno Hospital will be managed in an appropriate manner, anonymous through anonymized codes, in accordance with the provisions of current legislation on protection of personal data. In accordance with the same legislation for the data contained in the patient medical records, the patient’s authorization will be requested via a consent form. All data will be managed according to the Good Clinical Practice and the Good Pharmacovigilance practice referred to the observational studies (23, 24).

This retrospective observational project is based on the secondary use of data and the inclusion of adverse events in the RNF is not met. All the possible adverse events that will emerge from the extraction of the patients’ medical records and data analysis will be included in the final project report and since they are collected retrospectively, they will not be considered subjected to the reporting rules to the regulatory authority, as specified in the AIFA guidelines for Pharmacovigilance management.

All the variables extracted (**Supplementary Material**) from the patients ‘medical records will be organized in a file excel or data sheet according to the numerosity retrieved.
