# Supplementary material for: SodiuM glucose cotrANsporter-2 (SGLT-2) inhibitors in the treatment of type II DiAbetes in Tuscany: utiLizatiOn patteRns, and relatEd clinical use evaluation; the MANDALORE study protocol
Source: Front Endocrinol (Lausanne). 2026 Mar 23;17:1741084. doi: 10.3389/fendo.2026.1741084 (PMC13050743; doi:10.3389/fendo.2026.1741084)
Supplement: Supplementary file 1 [file DataSheet1.docx]

Supplementary Material

# Supplementary Data

**Variables will be extracted from patients’ medical records:**

1. Demographic Data

- Patient ID (anonymized)
- Age at the index date (first gliflozin prescription)
- Gender
- Date of birth (if available)
- Geographical residence
- Date of first prescription of the index drug (gliflozin)

1. Diagnosis and Clinical History

- Diagnosis of type 2 diabetes (date of first diabetes diagnosis, date of first visit, ICD-9 code)
- Comorbidities: (other reported medical conditions: ICD-9 code or description, date of first diagnosis for each comorbidity, if available)
- Treatment with other antidiabetic drugs (start date, discontinuation date, active ingredient, ATC code, medicinal product, dosage)
- History of cancer (type, ICD-9 code or description, and date of diagnosis)
- Treatment with concomitant medications for other conditions, as documented in the electronic medical records irrespective of prescription status (start date, discontinuation date, active ingredient, ATC code, medicinal product, dosage)

1. Treatment with Gliflozin

- Type of gliflozin prescribed: (active ingredient, medicinal product) (ATC gliflozin study in Box 1)
- Treatment line
- Type of therapy (monotherapy or combination therapy with metformin, gliptins, insulin, etc.)
- Date of initiation of therapy (date of first gliflozin prescription)
- Initial dosage (dose at the time of the first prescription)
- Treatment modifications (switching, drug change, and/or dose change)
- Date of switching and replacement drug
- Initial dose and final dose at switch
- Date of dose change
- Duration of therapy (dates of first prescription, dates of follow-up visits during therapy, date of end of therapy/discontinuation)
- Combination with other medications (concomitant medications, active ingredient, ATC code, medicinal product, dosage, date of visit at which they were recorded, date of initiation of concomitant medications)

1. Adverse Events

- Adverse events:
- Date of event
- Type of event (if serious, if it required hospitalization)
- Event description (ICD-9 code or descriptive)
- Outcome (improvement, resolution, complications)
- Date of resolution or outcome of adverse events (if documented)
- Management of the index drug following the adverse event (treatment discontinuation, dose reduction, continuation without modification, re-initiation; if documented)

1. Effectiveness

- HbA1c (levels recorded at all visits during the study period and date of each visit)
- eGFR (levels recorded at all visits during the study period and date of each visit)
- Fasting blood glucose (if available, levels recorded at all visits during the study period and date of each visit)
- Body weight (if monitored, levels recorded at all visits during the study period and date of each visit)
- Blood pressure (if available, levels recorded at all visits during the study period and date of each visit)

1. Follow-up

- Loss to follow-up: date and reason, if available

Box 1

| **ATC** | **Active substances** |
| --- | --- |
| A10BK01 | dapagliflozin |
| A10BD15 | dapagliflozin and metformin |
| A10BD25 | dapagliflozin, metformin, and saxagliptin |
| A10BD21 | dapagliflozin and saxagliptin |
| A10BD29 | dapagliflozin and sitagliptin |
| A10BK02 | canagliflozin |
| A10BD16 | canagliflozin and metformin |
| A10BK03 | empagliflozin |
| A10BD19 | empagliflozin and linagliptin |
| A10BD20 | empagliflozin and metformin |
| A10BD27 | empagliflozin, metformin and linagliptin |
| A10BK04 | ertugliflozin |
| A10BD23 | ertugliflozin and metformin |
| A10BD24 | ertugliflozin and sitagliptin |
